# Gut microbiota, physical activity and/or metabolic markers in healthy individuals - towards new biomarkers of health

**DOI:** 10.3389/fnut.2024.1438876

**Published:** 2024-11-28

**Authors:** Mari C. W. Myhrstad, Emilia Ruud, Line Gaundal, Terje Gjøvaag, Ida Rud, Kjetil Retterstøl, Stine M. Ulven, Kirsten B. Holven, Karsten Koehler, Vibeke H. Telle-Hansen

**Affiliations:** ^1^Department of Nursing and Health Promotion, Faculty of Health Sciences, Oslo Metropolitan University, Oslo, Norway; ^2^Nofima-Norwegian Institute of Food, Fisheries and Aquaculture Research, Ås, Norway; ^3^Department of Nutrition, Faculty of Medicine, Institute of Basic Medical Sciences, University of Oslo, Oslo, Norway; ^4^Norwegian National Advisory Unit on Familial Hypercholesterolemia, Department of Endocrinology, Morbid Obesity and Preventive Medicine, Oslo University Hospital Aker, Oslo, Norway; ^5^Department of Health and Sport Sciences, School of Medicine and Health, Technical University of Munich, Munich, Germany

**Keywords:** physical activity, gut microbiota, metabolic markers, aromatic amino acids, healthy individuals

## Abstract

**Background:**

The global prevalence of the metabolic disease Type 2 Diabetes (T2D) is increasing. Risk factors contributing to the development of T2D include overweight and obesity, lack of physical activity (PA), and an unhealthy diet. In addition, the gut microbiota has been shown to affect metabolic regulation. Since T2D is preventable, efforts should be put into the discovery of new biomarkers for early detection of individuals at risk of developing the disease.

**Objective:**

The objective of the cross-sectional study was to explore the relationship between gut microbiota and physical activity (PA) and/or metabolic markers such as selected amino acids (AA), markers of glycaemic regulation and lipid metabolism and anthropometric measures.

**Design:**

Healthy adults (18 and 65 years) with BMI between 18.5 and 27.5 kg/m2 originally recruited to a randomised controlled trial (RCT) (*n* = 17: six males, eleven females), were included in this exploratory cross-sectional study. Physical activity data was calculated based on a 3-days registration, and blood metabolome, gut microbiota analyses and anthropometric measures from one visit of the intervention were used in this cross-sectional study.

**Results:**

Of the 47 gut bacteria analysed, there were a total of 87 significant correlations with AA, PA, body composition and/or metabolic markers. Several of the gut bacteria correlated with both PA, metabolic or anthropometric markers.

**Conclusion:**

In this study, we demonstrate associations between gut bacteria and PA and/or metabolic markers including AA in healthy individuals. The results may guide future studies aiming at identifying new and early biomarkers of metabolic health and diseases.

## 1 Introduction

Globally, the prevalence of type 2 Diabetes (T2D) has increased dramatically over the last decades (1). T2D increases the risk of cardiovascular disease (CVD) and premature death and is therefore recognized as a major public health challenge (2). T2D is a metabolic disease and risk factors contributing to the development of T2D includes overweight and obesity, physical inactivity, and an unhealthy diet (3). T2D is preventable and given its global challenge, efforts should be put into the discovery of new biomarkers and early detection of individuals at risk of developing the disease.

In this regard, several new biomarkers of T2D risk have been suggested. These includes branched chain amino acids (BCAA) and aromatic amino acids (AAA) (4, 5). Besides being fundamental for protein synthesis, evidence indicate that these amino acids (AA) play key roles in signaling pathways and metabolic regulation. Circulating levels of BCAA (valine, leucin and isoleucine) and AAA (tyrosine and phenylalanine) have been positively correlated with insulin resistance in people with T2D (6, 7). In addition, tyrosine has been identified as a biomarker of glucose homeostasis and is significantly associated with incidents of T2D (8). The biological mechanisms explaining the relationship between these metabolites and T2D risk is not well described in the literature. It is suggested that BCAA may act as signaling molecules and may impact both glycemic regulation and lipid metabolism (9). In addition, increased circulating levels of BCAA have been shown to trigger mitochondrial dysfunction and thereby impact overall metabolic regulation (10). AA including tyrosine and phenylalanine are also biochemical precursors of neurotransmitters such as the catecholamines dopamine and norepinephrine, which are known to impact metabolic regulation (11).

Moderate physical activity (PA) is known to reduce the risk of T2D (12). This is linked to several mechanisms, including a positive impact on metabolic regulation and improved insulin sensitivity, reduced chronic low-grade inflammation, and reduced oxidative stress (13). In addition, PA is widely known as a behavioral factor that can influence body weight and is therefore indirectly important in management and prevention of metabolic diseases associated with obesity (14). The health beneficial effects of PA may also be linked to alterations in the gut microbiota (15), as highlighted by a recent systematic review showing that level of PA influenced the gut microbiota in humans (16).

The human gut microbiota are defined as the entire collection of microbes (archaea, bacteria, eukarya and viruses) living as a complex ecosystem in our gastrointestinal tract (17). Microorganisms in the gut microbiota play a protective and structural role in the intestinal mucosa. The main taxa of bacteria represented are Actinobacteria, Bacteroidetes, Cyanobacteria, Firmicutes, Fusobacteria, Proteobacteria and Verrucomicrobia, and about 90% of the bacterial flora in the gut are represented by Firmicutes and Bacteroidetes (18).

Gut microbes can affect the homeostasis of the host by producing AA, vitamins, and short-chain fatty acids (SCFA) (19). Short chain fatty acids may play a major role in host energy and substrate metabolism and are especially linked to improved metabolic regulation and may thereby reduce the risk of T2D (20, 21). Furthermore, recent studies have indicated a possible influence of PA on composition and function of the gut microbiota (22, 23). People exercising daily show higher levels of Firmicutes (24). Increased abundance of Firmicutes compared to Bacteroidetes has also been shown in the microbiota of athletes compared to sedentary controls (25). There are several suggested mechanisms to explain the interaction between PA and gut microbiota. Exercise may affect gut motility, integrity and mobility that may lead to reduced transit time, physical changes in the gastrointestinal tract including pH, mucus secretion and nutrients availability (26). All of which may affect the gut microbiota composition. It is interesting to note that physical activity has been linked to alter the gut microbial composition towards increased capacity to produce SCFAs (27, 28). Furthermore, exercise and gut microbiota may positively affect health through the synthesis and release of neurotransmitters from the gut (29). The potential mechanism is not fully elucidated but may be linked to metabolization of AA by the gut microbiota (30). Whether or not changes in the gut microbiota caused by PA is associated with beneficial effects on metabolic markers and will impact health over time is however, currently not known.

Towards the goal of identifying new and early biomarkers of metabolic diseases, we investigated the relationship between gut microbiota PA, metabolic markers and anthropometric measures, in a cross-sectional study in healthy individuals. The metabolic markers include markers related to different aspects of the metabolism such as glycemic regulation, lipid- and AA metabolism.

## 2 Materials and method

### 2.1 Subjects and study design

The current cross-sectional study is a secondary analysis of a randomised controlled dietary crossover study, with the primary aim to investigate the short-term effect of replacing dietary saturated fatty acids with polyunsaturated fatty acids on glycaemic regulation (31). In the current study, data from one visit were used and healthy adults (18–65 years) with BMI between 18.5 and 27.5 kg/m^2^ (*n* = 17: six males, eleven females) (31), were included. At this visit, blood samples were collected, and anthropometric measures were performed as further described below. The participants were instructed to wear the accelerometer for 3 days. Data from these 3 days were used to calculate the PA (mean values of the 3 days) as further described below. In addition, the participants were instructed to collect a stool sample before the visit and bring the sample to the research lab at the visit. An overview of the included variables can be found in Supplementary Table S1. The participants were recruited from the student body and employees at Oslo Metropolitan University (OsloMet) and through advertisement on Facebook and OsloMet website. Participants had to be willing to limit their intake of dietary fats and products rich in beta-glucan 1 week prior to and during the study. Use of dietary supplements or probiotic products were not allowed 4 weeks prior to and during the study. Apart from that, participants were advised to maintain their habitual diet and PA level throughout the study.

Exclusion criteria were fasting blood glucose values ≥ 6,1 mmoL/L, micro C-reactive protein (mCRP) > 10 mg/L, chronic diseases (e. g. diabetes, CVD and cancer), intestinal diseases (e. g. inflammatory bowel diseases, celiac disease and irritable bowel disease) and food allergies or intolerances. Further exclusion criteria were antibiotic treatment during the previous 3 months as well as throughout the study, blood donations during the previous 2 months and throughout the study, pregnancy, or lactation, planned weight reduction, 5% weight change the previous 3 months, alcohol consumption >40 g/d, use of tobacco and hormonal treatment (except for oral contraception).

The study was conducted according to the guidelines laid down in the Declaration of Helsinki, and all procedures involving human subjects were approved by the Regional Committees for Medical and Health Research Ethics (REK) (approval #2018/104). A biobank with REK approval (2018/104) is used for storage of biological materials, according to Norwegian law. Written informed consent was obtained from all subjects. The original RCT study was registered at Clinical Trials (https://clinicaltrials.gov/, registration identification number: NCT03658681).

### 2.2 Blood sampling and clinical assessment

Blood sampling and clinical assessment were collected after an overnight fast (≥ 12 h). The participants were instructed to refrain from alcohol consumption and excessive PA the day before blood sampling. Glucose was measured with a HemoCue Glucose 201 Analyser and Micro cuvettes following a standardised procedure. The HemoCue 201 Micro cuvettes were stored in a refrigerator (4°C) and taken out in room temperature 30 min prior to blood sampling. Triglycerides (TG), Hemoglobin A1c (HbA1c) and insulin were analysed in serum obtained from 8.5 mL serum gel tubes and turned 6–10 times before spin down after 30 min (1,300–1,500 g, 15 min). Serum was kept in a refrigerator (4°C) before it was sent to a routine laboratory (Fürst Medical Laboratory) within 24 h. Non-esterified fatty acids (NEFA) was measured using an enzymatic colorimetric assay and the SCFA acetate, propionate and butyrate were measured in EDTA plasma. Both analyses were performed in a commercial laboratory (Vitas Analytical Service, Oslo, Norway). Homeostasis model assessment-insulin resistance (HOMA-IR) and Matsuda index was calculated as previously described (31).

### 2.3 Metabolome analysis: NMR spectroscopy

The fasting plasma samples goes through a fully automated nuclear magnetic resonance (NMR) analysis process by Nightingale (Nightingale Health), whereby individual analytes within a sample are separated by their magnetic resonance shift or mass/charge ratio, resulting in a separated spectral profile (32). The metabolome data comprised of 250 variables including metabolic measures. In this study we used variables related to metabolic regulation and physical activity including lactate, citrate, and AA (alanine, glutamine, histidine, isoleucine, leucine, phenylalanine, tyrosine, and valine).

### 2.4 Anthropometry

Body weight and composition were measured after an overnight fast using a bioelectrical impedance scale (Tanita BC-418 Segmental Body Composition Analyser). Any metal (i.e., belt, jewellery, watch etc.), shoes and socks (33) were removed before the measurement. One kg was subtracted from the body weight to compensate for clothing. Height was measured by a wall-mounted stadiometer.

### 2.5 Food frequency questionnaire

Participants completed a validated food frequency questionnaire (FFQ) before study start, reflecting dietary intake for the past 12 months (33). The FFQ consisted of 270 food items and included questions about frequency of intake (from never to several times a day) and portions size based on household units (cups, glasses, slices, pieces, spoons, and teaspoons). Food groups such as grain products, vegetables, fruit, berries, nuts, seeds, meat, fish, cheese, butter, margarine and oils were included in the FFQ (33). Information about consumption of macronutrients and fibre was used to assess correlation with the gut microbiota and metabolic and anthropometric markers.

### 2.6 Faecal collection and gut microbiota analyses

Participants received a sample collection kit (GA-map™ Dysbiosis Test, Genetic Analysis AS) for faecal collection and were instructed to sample the stool from three different places and place it in the sampling tubes. The samples were kept at room temperature for a maximum of 3 days before they were stored at −80°C at OsloMet. Participants were instructed to sample the stool as close to the visit as possible. All samples were collectively sent to Genetic Analysis AS (GA) for microbiota analyses after the study was completed. The GA-map™ Dysbiosis Test is a commercially available genome-based test using faecal samples for analyses of gut bacteria, described in detail elsewhere (34). The test comprises 48 deoxyribonucleic acid (DNA) probes targeting ≥300 bacteria on different taxonomic levels, thus allowing mapping of the intestinal microbiota profile for a selected set of bacteria. To characterize and identify bacteria present, probes targeting seven variable regions (V3 – V9) of the 16S rRNA gene were used. Human faecal sample homogenization, mechanical and enzymatic bacterial cell disruption was utilized to isolate and bind total bacterial genomic DNA to magnetic beads. The hypervariable regions V3 – V9 of the 16S rRNA were further amplified by polymerase chain reaction (PCR). To determine bacterial DNA labelling, single nucleotide extension and hybridization to a complementary DNA strand was coupled to beads. Abundance of bacteria was assessed by the strength of fluorescent signal (probe intensity) and measured by Luminex 200 (Luminex Corporation). Forty-seven bacteria were included in the analyses.

### 2.7 Physical activity

PA was measured throughout the study period using a three-axial accelerometer (ActiGraph GTX3, ActiGraph Corporation). Participants were instructed to wear the accelerometer at the left hip during each day of the study except from situations with water-contact, such as swimming and showering, and during sleep. After the data collection period, the data were downloaded using the ActiLife 6 sotware (ActiGraph Corporation). Wear and non-wear-time classification was performed according to Choi et al. (35), and wear-time validated data were summarised into daily average counts. For each participant, the same three weekdays (no weekend days) were used. For one participant the accelerometer was used for <10 h/d and PA was not satisfactory monitored. Hence, for the analysis of PA, the number of participants is sixteen. Furthermore, Freedson three-axis vector magnitude accelerometer count threshold values were used to characterize accelerometer counts as percentage time spent in light (0–2,690 cpm), moderate (2,691–6,166 cpm), vigorous (6,167–9,642 cpm) and very vigorous intensity PA (≥9,643 cpm). Parameters of PA used for the analysis were time spent in moderate to vigorous PA (MVPA) (min), total sedentary bouts (min) and total steps. Average of 3 days was used in the current correlation analyses.

### 2.8 Statistical analysis

Gut bacteria were log2-transformed before analysis. Correlations between PA-variables, metabolic markers, anthropometric measures, and gut bacteria were assessed with Spearman rank correlation. All the included variables are listed in Supplementary Table S1. Only bacteria and AA showing statistically significant correlations with correlations coefficients are presented. *p* < 0.05 was regarded as statistically significant. Considering the explorative nature of the present study, correction for multiple testing was not performed. All statistical analyses were performed in IBM SPSS statistics (version 28.0.1.1) after processing the data in Microsoft Excel for Windows (16.0.16026.20002). Figures were created using BioRender.com and GraphPad Prism.

## 3 Results

### 3.1 Baseline characteristics

Seventeen healthy participants (six males, eleven females) were included in this cross-sectional study. The baseline characteristics of the participants have been previously reported and are shown in Table 1 (31). The participants were healthy adults (median age 28 years). Fasting levels of blood glucose, HbA1c, insulin, total cholesterol, TG, and systolic and diastolic blood pressure were within the normal range (Table 1) (31). The participants had a median BMI of 22.8 (25th – 75th percentiles: 22.0–25.0). Level of daily PA was relatively high with a median of 247.4 min in MVPA (25th – 75th percentiles: 185.8–311.3) and total steps of 25654.5 (25th – 75th percentiles: 19649.3–29906.8) (Table 1).

**Table 1:** Baseline characteristics of the participants (median and 25th–75th percentiles) (31).

	Median (25th–75th percentiles)
Male/female (*n*)	6/11
Age (years)	28.0 (25.0–46.0)
**Metabolic risk markers**	
HbA1c (%)^a^	5.2 (5.0–5.4)
HbA1c (mmol/mol)^a^	33.0 (31.2–35.5)
Glucose (mmol/l)	5.1 (4.9–5.4)
Insulin (pmol/l)	51.0 (31.0–60.0)
TG (mmol/l)	0.9 (0.6–1.4)
Total Cholesterol (mmol/l)	4.9 (4.4–5.4)
mCRP (mg/l)	0.9 (0.3–1.4)
Systolic blood pressure (mmHg)^a^	123 (113–136)
Diastolic blood pressure (mmHg)^a^	71 (66–75)
**Anthropometric measurements**	
Body fat (%)	24.4 (12.5–33.9)
BMI (kg/m^2^)	22.8 (22.0–25.0)
**Diet** ^b^	
Protein (E%)	15.7 (14.0–16.5)
Fats (E%)	34.8 (31.4–36.5)
Carbohydrates (E%)	43.8 (39.3–47.3)
Fibre (g)	39.6 (31.8–51.7)
**Physical activity** ^c^	
MVPA (min)	247.4 (39.3–47.3)
Total sedentary bouts (min)	32.0 (21.5–44.8)
Total steps	25654.5 (19649.3–29906.8)

mCRP, micro C-reactive protein; FFM, fat-free mass; HbA1c, hemoglobin A1c; TG, triglycerides; MVPA, moderate to vigorous physical activity.

a HbA1c, systolic and diastolic blood pressure measured at screening.

b As previously shown by Gaundal et al. through a FFQ.

c MVPA, total sedentary bouts and total steps includes data from 3 days. Rest of the variables were measured at baseline. All variables are measured fasted.

### 3.2 Gut bacteria, physical activity, and metabolic markers

The abundance of the 47 gut bacteria included in this study represents bacteria belonging to the phyla Actinobacteria, Bacteroidetes, Firmicutes, Mycoplasmatota, Proteobacteria and Verrucomicrobia.

**Figure 1:**
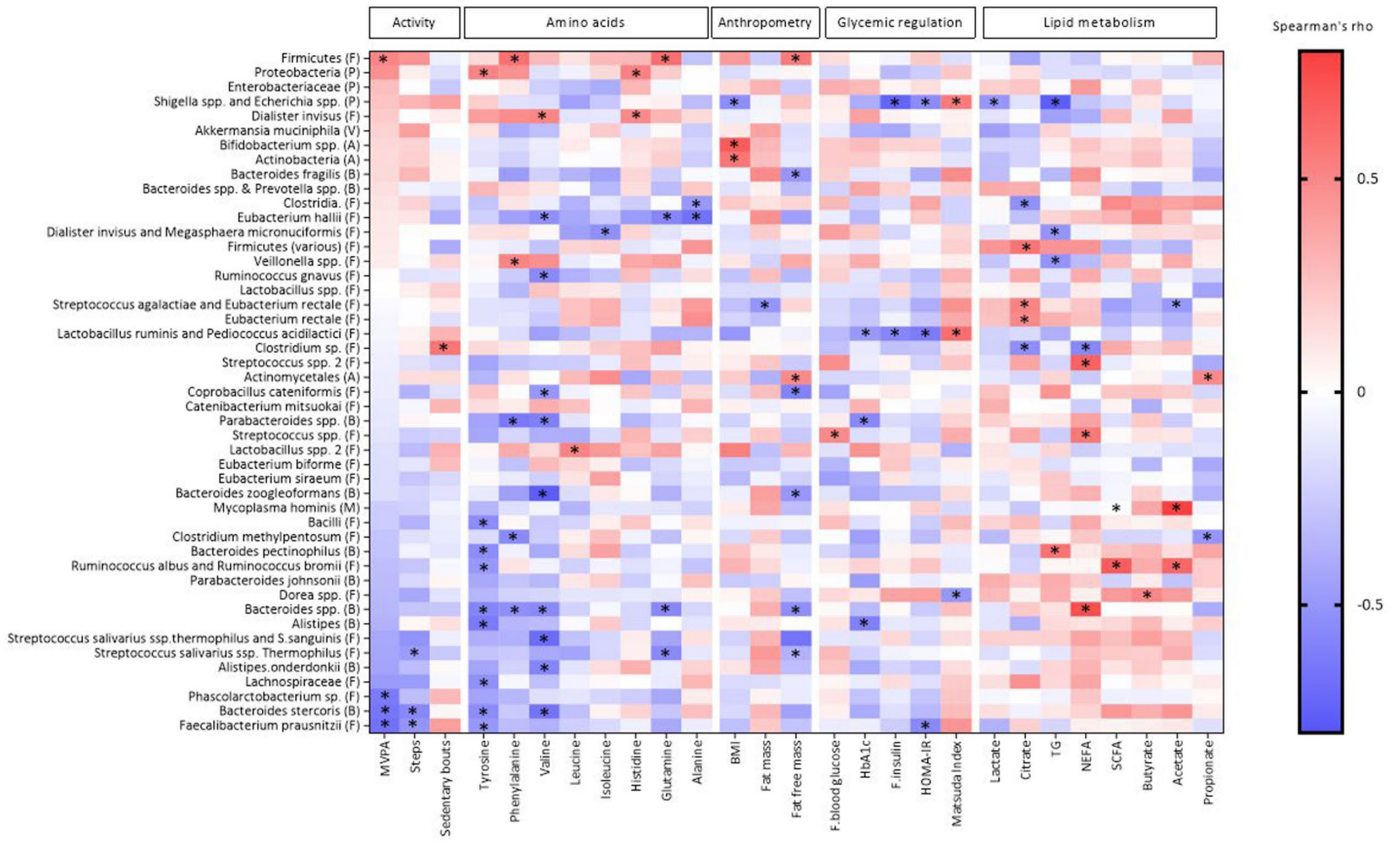
Heat map of Spearman’s coefficient between gut bacteria, metabolic markers, physical activity and anthropometric indicators. The bacterial taxa are sorted from negative (blue) to positive (red) correlations in relation to the amount of MVPA (min) assessed by Spearman’s correlation. Significant correlations are marked by *. Phylum is indicated within parentheses; A, actinobacteria; B, bacteroidetes; F, firmicutes; P, proteobacteria; M, mycoplasmatota; V, verrucomicrobia; HbA1c, hemoglobin A1c; TG, triglycerides; NEFA, non-esterified fatty acids; SCFA, short-chain fatty acids; HOMA-IR, homeostasis model assessment-insulin resistance. Created by BioRender.com.

We found 87 significant correlations between the bacteria and AA, PA, body composition, or metabolic markers shown in Figure 1 and Supplementary Table S1. There were 34 significant correlations between the bacteria and the AA, eight significant correlations between the bacteria and PA and 45 significant correlations between the bacteria and anthropometric and metabolic measures (Figure 1; Supplementary Table S2).

Of the eight AA included from the NMR metabolome analyses (see method section), tyrosine was correlated with nine bacteria, of which eight were negatively correlated (*Alistipes, Bacteroides pectinophilus, Bacteroides* spp.*, Bacteriodes stercoris, Bacilli, Faecalibacterium prausnitzii, Lachnospiraceae, Ruminococcus albus and Ruminococcus bromii*) and one positively correlated (*Proteobacteria*). Valine was correlated with ten bacteria, of which nine were negatively correlated (*Alistipes onderdonkii, Bacteroides* spp.*, Bacteroides stercoris, Bacteroides zoogleoformans, Parabacteroides* spp.*, Coprobacillus cateniformis, Eubacterium hallii, Ruminococcus gnavus, and Streptococcus salivarius ssp. thermophilus and S. sanguinis*) and one positively correlated (*Dialister invisus*). Phenylalanine was correlated with five bacteria, of which three negatively (*Bacteroides* spp.*, Parabacteroides* spp.*, Clostridium methylpentosum*) and two positively (Firmicutes and *Veillonella* spp.). Alanine was negatively correlated with two bacteria: *Clostridia. and Eubacterium hallii*. Glutamine was correlated with four bacteria, of which three negatively (*Bacteroides* spp.*, Eubacterium hallii and Streptococcus salivarius ssp. Thermophilus*) and one positively (Firmicutes). Histamine was correlated with two bacteria, of which one was negatively (*Dialister invisus*) and one positively (*Proteobacteria*). Isoleucine was negatively correlated with *Dialister invisus and Megasphaera micronuciformis*, while Leucine was positively correlated with *Lactobacillus* spp. *2*. All correlations are shown in Figure 1 and correlation coefficients and *p*-values can be found in Supplementary Table S1.

PA measured as three different parameters including MVPA, total sedentary bouts and total steps, was correlated with eight bacteria (Figure 1; Supplementary Table S1). MVPA was negatively correlated with three bacteria (*Bacteroides stercoris, Faecalibacterium prausnitzii and Phascolarctobacterium* sp.), and was positively correlated with Firmicutes. The number of sedentary bouts was positively correlated with *Clostridium* sp. Number of steps was negatively correlated with *Bacteroides stercoris* and *Faecalibacterium prausnitzii*.

**Figure 2:**
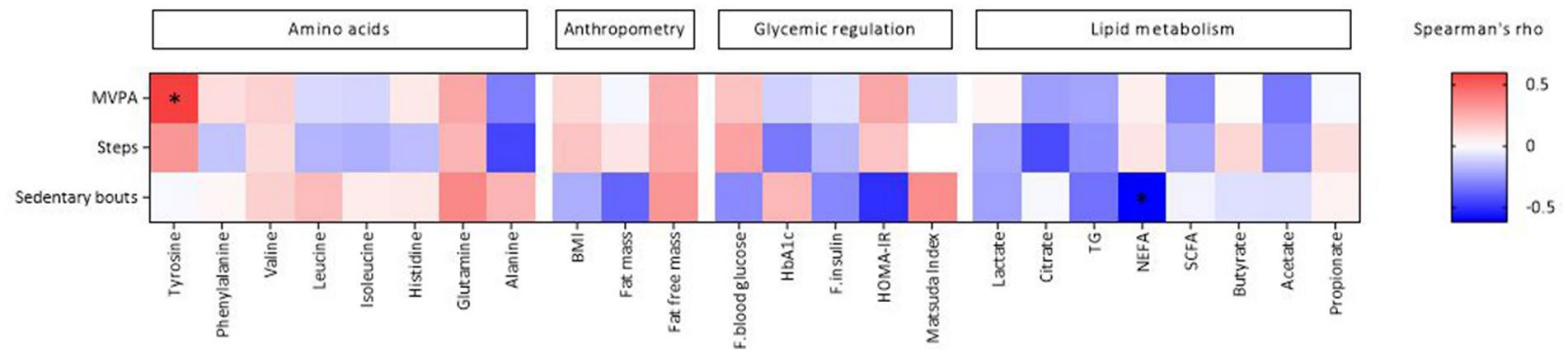
Heat map of Spearman’s coefficient between physical activity and metabolic and anthropometric markers. Physical activity is sorted from negative (blue) to positive (red) correlations in relation to the amount of tyrosine assessed by spearman’s correlation. Significant correlations are marked by *. HbA1c, hemoglobin A1c; TG, triglycerides; NEFA, non-esterified fatty acids; SCFA, short-chain fatty acids; HOMA-IR, homeostasis model assessment-insulin resistance. Created by BioRender.com.

Furthermore, MVPA was positively correlated with the AAA tyrosine, while sedentary bouts were negatively correlated with NEFA (Figure 2; Supplementary Table S1).

## 4 Discussion

In this study, we explored the relationship between gut bacteria, and PA, and/or metabolic markers using cross-sectional data from healthy, physical active adults. Of the 47 gut bacteria analysed in this study, there were a total of 87 significant correlations with AA, PA, body composition or metabolic markers.

We found that several of the measured gut bacteria were significantly correlated with indicators of PA. This is in line with several other studies. Zhao et al. found that exercise induced changes in the gut microbiota (36), and in a recent paper by Wang and colleagues they summarized the connections between gut microbiota and musculoskeletal health (26). Furthermore, mice lacking gut microbiota (germ-free mice) had lower skeletal muscle mass than normal germ-carrying mice (37), suggesting that a given gut microbiota may be important for musculoskeletal health.

We found a positive correlation between MVPA and levels of Firmicutes. Other studies also reported higher levels of Firmicutes in physical active when compared to inactive individuals (24, 28, 38). Increased abundance of Firmicutes compared to Bacteroidetes has been shown in the microbiota of athletes compared to sedentary controls (25). In a study by Squillario et al., the faecal microbiome of obese and normal weight individuals was analysed and the Firmicutes/Bacteroidetes (F/B) ratio in obese individuals was lower than the normal weight individuals (39). Given these results, it would be interesting to look further into the Firmicutes phylum and possible relation to physical activity and sedentary lifestyle.

We found a negative correlation between MVPA and steps and *Faecalibacterium prausnitzii*, belonging to the Firmicutes phylum. Allen et al. explored the effects of exercise on gut microbiota after a 6-weeks supervised endurance training program in lean and obese sedentary adults (40). Lean sedentary adults achieved increased amount of *Faecalibacterium* species and decreased amount of Bacteroidetes species after endurance training, while the contrary was registered in obese sedentary adults (40). Participants in our study were healthy with mainly normal weight and PA was only measured for 3 days. Therefore, future studies should aim to compare the effect of PA for a longer time in both healthy people and people with metabolic dysregulation to better understand the role of the *Faecalibacterium* species in metabolic regulation.

Even though we and others have found a negative correlation between PA and *Faecalibacterium prausnitzii*, indicating low levels of the bacteria to be beneficial, other studies have found that high levels of the bacteria may be health beneficial (41). In studies investigating gut bacteria and Chron’s disease, *Faecalibacterium prausnitzii* has been shown to have positive health benefits by exhibit anti-inflammatory effects, as reduction of the bacteria is associated with a higher risk of postoperative recurrence of Chron’s disease (42). In addition, levels of *Faecalibacterium prausnitzii* increased after intake of a health promoting mediterranean diet (43). The discrepancies may be attributed to different study design including participants and the health outcome measured. Whether *Faecalibacterium prausnitzii* may be regarded a health beneficial microbe is therefore currently not known.

In our study, we also found a negative correlation between MVPA and steps and *Bacteroides stercoris* which belongs to the Bacteroidetes phylum. Bacteroidetes are involved in food digestion, gut environmental control, signal transmission and inhibiting growth of detrimental microorganisms in the gut (44). However, a high abundance of this phylum is associated with poor microbiota with low diversity (44). *Bacteroides stercoris* was significantly enriched in faecal samples from patients with diabetic nephropathy (45) and was higher in the Ulcerative Colitis group than in the healthy donor group (46). Furthermore, Bacteroidetes have been shown to be positively correlated with obesity and BMI (39, 47). This is in line with our results showing a lower level of *Bacteroides stercoris* with higher amounts of PA (measured as MVPA and steps). Taken together, this may indicate *Bacteroides stercoris* as a new biomarker for health.

In our study, the AAA tyrosine level in blood was positively correlated with MVPA. Tyrosine and MVPA were both negatively correlated with both *Faecalibacterium prausnitzii* and *Bacteroides stercoris*. Tyrosine has been suggested as a new biomarker of insulin resistance and T2D (4, 5), in which high levels have been suggested to be unbeneficial. This is contrary to our results showing that tyrosine positively correlated with MVPA. Our results are supported by a study by Strasser et al. investigating the effect of probiotics and exhaustive exercise on AA levels, who found increased levels of the tyrosine after exercising (48). Zhao et al. also found that exercise induced changes in the gut microbiota and the faecal metabolome (36). Metabolites from faeces were analysed before and after running a half marathon. Compared to the faecal metabolite profile before running, phenylalanine, tyrosine and tryptophan biosynthesis pathway increased, while there were no significant differences in gut microbiota diversity after running. However, they observed more bacterial taxa ranking from the phylum to species levels after running than before running, indicating that running potentially increased the diversity of the gut microbiota (36). Similar results were also observed in a study from Tabone et al. A single bout of exercise induced changes in the microbiota and increased the level of tryptophan, tyrosine, and phenylalanine metabolites in faeces (49). Whether or not PA, tyrosine and the discussed gut bacteria is causally related and will impact and predict a future health response, needs further investigations.

### 4.1 Limitations, strengths, and future perspectives

The current study has several limitations. It is a cross-sectional study, and no causal relationship can therefore be drawn. The study has few participants and the PA registrations was only conducted for 3 days. Correlation analyses without considering multiple testing have been conducted, due to the explorative nature of the study. Furthermore, there are several factors that will shape a person’s gut microbiota, including diet. In the current analyses we did not adjust for any confounding factors due to the relative low number of participants. In addition, the microbiota analyses were targeted, meaning that only selected bacteria were included in the analyses. The results should therefore be interpretated with caution but may give valuable directions for future studies. There are several RCTs and systematic reviews that have investigated the effect of PA on gut microbiota (16, 50). However, whether the alterations in gut microbiota will impact health have to our knowledge, not been properly addressed. We suggest that future studies may have a focus on causal relationships. RCTs with more participants and a long-term PA intervention, including dietary registration, effect on gut microbiota, and metabolic markers, are needed to investigate any causal relationship. Future studies should address the effect of PA on metabolic risk markers in general or may focus on specific biomarkers such as AA. It is also of interest to investigate the potential differences in the duration and intensity of PA on gut microbiota and metabolic risk factors. Lastly, targeting the gut microbiota with diet such as fibre may also increase the health beneficial effects of PA and should be further explored. The strengths of the study are the high number of metabolites and gut bacteria analysed, and the continuous measures of PA using the current gold-standard for objective PA assessment.

## 5 Conclusion

In this study, we demonstrate associations between gut bacteria and PA and/or metabolic markers including AA in healthy individuals. We suggest that gut bacteria may be used as future biomarkers of PA and in early detection of metabolic diseases. However, duration and intensity of PA may have different effects on gut bacteria, and hence different impact on health. The current results may guide directions for future studies investigating causal relationship between gut bacteria, PA, and health outcomes.

## Data Availability

The datasets presented in this article are not readily available because of privacy restrictions. Requests to access the datasets should be directed to corresponding author.
