# Supplementary material for: Gut microbiota, physical activity and/or metabolic markers in healthy individuals - towards new biomarkers of health
Source: Front Nutr. 2024 Nov 28;11:1438876. doi: 10.3389/fnut.2024.1438876 (PMC11635997; doi:10.3389/fnut.2024.1438876)
Supplement: Supplementary file 1 [file Table_1.DOCX]

**Supplementary Table 1.** Overview of variables used in analyses including, metabolic and anthropometric markers, markers of physical activity and gut bacteria

| **Groups of Variables** | **Varibables** | **Platform and/or site of analyses** |
| --- | --- | --- |
| *Amino acids* | Tyrosine | NMR spectroscopy, Nightingale |
|  | Phenylalanine | NMR spectroscopy, Nightingale |
|  | Valine | NMR spectroscopy, Nightingale |
|  | Leucine | NMR spectroscopy, Nightingale |
|  | Isoleucine | NMR spectroscopy, Nightingale |
|  | Histidine | NMR spectroscopy, Nightingale |
|  | Glutamine | NMR spectroscopy, Nightingale |
|  | Alanine | NMR spectroscopy, Nightingale |
| *Anthropometry* | BMI | Calculated |
|  | Fatt mass | Bioelectrical impedance Tanita BC-418 |
|  | Fat free mass | Bioelectrical impedance Tanita BC-418 |
| *Glycemic regulation* | Fasting blood glucose | HemoCue analyser and micro cuvette |
|  | HbA1c | Fürst Medical Laboratory |
|  | Fasting Insulin | Fürst Medical Laboratory |
|  | Homa IR (Insulin resistance) | Calculated |
|  | Matsuda Index (Insulin sensitivity) | Calculated |
| *Lipid metabolism* | Lactate | NMR spectroscopy, Nightingale |
|  | Citrate | NMR spectroscopy, Nightingale |
|  | Triglycerides | Enzymatic colorimetric assay, Fürst Medical Laboratory |
|  | NEFA | Vitas Analytical Service |
|  | SCFA (Total level of Butyrate, acetate, and propionate) | Vitas Analytical Service |
|  | Butyrate | Vitas Analytical Service |
|  | Acetate | Vitas Analytical Service |
|  | Propionate | Vitas Analytical Service |
| *Physical activity* | MVPA | Wearable sensor, ActiGraph GTX3 |
|  | Total sedentary bouts | Wearable sensor, ActiGraph GTX |
|  | Total steps | Wearable sensor, ActiGraph GTX3 |
| *Gut bacteria* | Actinobacteria | GA-map™ Dysbiosis Test, Genetic Analysis |
|  | Actinomycetales | GA-map™ Dysbiosis Test, Genetic Analysis |
|  | Bifidobacterium spp. | GA-map™ Dysbiosis Test, Genetic Analysis |
|  | Alistipes | GA-map™ Dysbiosis Test, Genetic Analysis |
|  | Alistipes onderdonkii | GA-map™ Dysbiosis Test, Genetic Analysis |
|  | Bacteroides fragilis | GA-map™ Dysbiosis Test, Genetic Analysis |
|  | Bacteroides pectinophilus | GA-map™ Dysbiosis Test, Genetic Analysis |
|  | Bacteroides spp. | GA-map™ Dysbiosis Test, Genetic Analysis |
|  | Bacteroides spp. & Prevotella spp. | GA-map™ Dysbiosis Test, Genetic Analysis |
|  | Bacteroides stercoris | GA-map™ Dysbiosis Test, Genetic Analysis |
|  | Bacteroides zoogleoformans | GA-map™ Dysbiosis Test, Genetic Analysis |
|  | Parabacteroides johnsonii | GA-map™ Dysbiosis Test, Genetic Analysis |
|  | Parabacteroides spp. | GA-map™ Dysbiosis Test, Genetic Analysis |
|  | Firmicutes | GA-map™ Dysbiosis Test, Genetic Analysis |
|  | Bacilli | GA-map™ Dysbiosis Test, Genetic Analysis |
|  | Catenibacterium mitsuokai | GA-map™ Dysbiosis Test, Genetic Analysis |
|  | Clostridia | GA-map™ Dysbiosis Test, Genetic Analysis |
|  | Clostridium methylpentosum | GA-map™ Dysbiosis Test, Genetic Analysis |
|  | Clostridium spp. | GA-map™ Dysbiosis Test, Genetic Analysis |
|  | Coprobacillus cateniformis | GA-map™ Dysbiosis Test, Genetic Analysis |
|  | Dialister invisus | GA-map™ Dysbiosis Test, Genetic Analysis |
|  | Dialister invisus and Megasphaera micronuciformis | GA-map™ Dysbiosis Test, Genetic Analysis |
|  | Dorea spp. | GA-map™ Dysbiosis Test, Genetic Analysis |
|  | Eubacterium biforme | GA-map™ Dysbiosis Test, Genetic Analysis |
|  | Eubacterium hallii | GA-map™ Dysbiosis Test, Genetic Analysis |
|  | Eubacterium rectale | GA-map™ Dysbiosis Test, Genetic Analysis |
|  | Eubacterium siraeum | GA-map™ Dysbiosis Test, Genetic Analysis |
|  | Faecalibacterium prausnitzii | GA-map™ Dysbiosis Test, Genetic Analysis |
|  | Lachnospiraceae | GA-map™ Dysbiosis Test, Genetic Analysis |
|  | Lactobacillus ruminis and Pediococcus acidilactici | GA-map™ Dysbiosis Test, Genetic Analysis |
|  | Lactobacillus spp. | GA-map™ Dysbiosis Test, Genetic Analysis |
|  | Lactobacillus spp. 2 | GA-map™ Dysbiosis Test, Genetic Analysis |
|  | Phascolarctobacterium spp. | GA-map™ Dysbiosis Test, Genetic Analysis |
|  | Ruminococcus albus and Ruminococcus bromii | GA-map™ Dysbiosis Test, Genetic Analysis |
|  | Ruminococcus gnavus | GA-map™ Dysbiosis Test, Genetic Analysis |
|  | Streptococcus agalactiae and Eubacterium rectale | GA-map™ Dysbiosis Test, Genetic Analysis |
|  | Streptococcus salivarius ssp.thermophilus and S.sanguinis | GA-map™ Dysbiosis Test, Genetic Analysis |
|  | Streptococcus salivarius ssp. Thermophilus | GA-map™ Dysbiosis Test, Genetic Analysis |
|  | Streptococcus spp. | GA-map™ Dysbiosis Test, Genetic Analysis |
|  | Streptococcus spp. 2 | GA-map™ Dysbiosis Test, Genetic Analysis |
|  | Veillonella spp. | GA-map™ Dysbiosis Test, Genetic Analysis |
|  | Firmicutes (various) | GA-map™ Dysbiosis Test, Genetic Analysis |
|  | Proteobacteria | GA-map™ Dysbiosis Test, Genetic Analysis |
|  | Enterobacteriaceae | GA-map™ Dysbiosis Test, Genetic Analysis |
|  | Shigella spp. and Echerichia spp. | GA-map™ Dysbiosis Test, Genetic Analysis |
|  | Mycoplasma hominis | GA-map™ Dysbiosis Test, Genetic Analysis |
|  | Akkermansia muciniphila | GA-map™ Dysbiosis Test, Genetic Analysis |

BMI: Body mass index, MVPA: Moderate to vigorous physical activity, NEFA: Non-esterified fatty acids, SCFA: Short chain fatty acid, HbA1c: Glycated hemoglobin
