# Supplementary material for: Gut microbiota, physical activity and/or metabolic markers in healthy individuals - towards new biomarkers of health
Source: Front Nutr. 2024 Nov 28;11:1438876. doi: 10.3389/fnut.2024.1438876 (PMC11635997; doi:10.3389/fnut.2024.1438876)
Supplement: Supplementary file 2 [file Table_2.DOCX]

**Supplementary Table 2.** Correlations between metabolic and physical activity variables and gut bacteria were assessed with Spearman rank correlation and only significant correlations (P-value < 0.05) are given in the Table.

|  | Correlation coefficient | P-value |
| --- | --- | --- |
| Tyrosine |  |  |
| Alistipes | -0.630 | 0.007 |
| Bacteroides pectinophilus | -0.543 | 0.024 |
| Bacteroides spp. | -0.578 | 0.015 |
| Bacteroides stercoris | -0.543 | 0.024 |
| Bacilli | -0.543 | 0.024 |
| Faecalibacterium prausnitzii | -0.490 | 0.046 |
| Lachnospiraceae | -0.525 | 0.030 |
| Ruminococcus albus and Ruminococcus bromii | -0.493 | 0.044 |
| Proteobacteria | 0.508 | 0.038 |
| Phenylalanine |  |  |
| Bacteroides spp. | -0.529 | 0.029 |
| Parabacteroides spp. | -0.635 | 0.006 |
| Clostridium methylpentosum | -0.556 | 0.021 |
| Firmicutes | 0.589 | 0.013 |
| Veillonella spp. | 0.513 | 0.035 |
| Valine |  |  |
| Alistipes.onderdonkii | -0.577 | 0.015 |
| Bacteroides spp. | -0.599 | 0.020 |
| Bacteroides stercoris | -0.658 | 0.004 |
| Bacteroides zoogleoformans | -0.768 | <0.001 |
| Parabacteroides spp. | -0.600 | 0.011 |
| Coprobacillus cateniformis | -0.492 | 0.045 |
| Eubacterium hallii | -0.531 | 0.028 |
| Ruminococcus gnavus | -0.561 | 0.019 |
| Streptococcus salivarius ssp.thermophilus and S.sanguinis | -0.698 | 0.002 |
| Dialister invisus | 0.502 | 0.040 |
| Leucine |  |  |
| Lactobacillus spp. 2 | 0.508 | 0.037 |
| Isoleucine |  |  |
| Dialister invisus and Megasphaera micronuciformis | -0.524 | 0.031 |
| Histidine |  |  |
| Dialister invisus | -0.487 | 0.048 |
| Proteobacteria | 0.523 | 0.031 |
| Glutamine |  |  |
| Bacteroides spp. | -0.564 | 0.018 |
| Eubacterium hallii | -0.575 | 0.016 |
| Streptococcus salivarius ssp. Thermophillus | -0.561 | 0.019 |
| Firmicutes | 0.595 | 0.012 |
| Alanine |  |  |
| Clostridia | -0.483 | 0.050 |
| Eubacterium hallii | -0.668 | 0.003 |
| MVPA |  |  |
| Bacteroides stercoris | -0.650 | 0.006 |
| Faecalibacterium prausnitzii | -0.674 | 0.004 |
| Phascolarctobacterium sp. | -0.629 | 0.009 |
| Firmicutes | 0.503 | 0.047 |
| Steps |  |  |
| Bacteroides stercoris | -0.562 | 0.024 |
| Faecalibacterium prausnitzii | -0.509 | 0.044 |
| Streptococcus salivarius ssp. Thermophillus | -0.519 | 0.039 |
| Sedentary bouts |  |  |
| Clostridium sp. | 0.574 | 0.020 |
| BMI |  |  |
| Actinobacteria | 0.517 | 0.034 |
| Bifidobacterium spp. | 0.625 | 0.007 |
| Shigella spp. and Echerichia spp. | -0.531 | 0.028 |
| Fat mass |  |  |
| Streptococcus agalactiae and Eubacterium rectale | -0.502 | 0.040 |
| Fat free mass |  |  |
| Actinomycetales | 0.489 | 0.046 |
| Bacteroides fragillis | -0.498 | 0.042 |
| Bacteroides spp. | -0.529 | 0.029 |
| Bacteroides zoogleoformans | -0.497 | 0.043 |
| Firmicutes | 0.548 | 0.023 |
| Coprpbacillus cateniformis | -0.583 | 0.014 |
| Streptococcus salivarius ssp. Thermophillus | -0.661 | 0.004 |
| Fasting blood glucose |  |  |
| Streptococcus spp. | 0.485 | 0.049 |
| HbA1c |  |  |
| Alistipes | -0.601 | 0.011 |
| Parabacteroides spp. | -0.563 | 0.019 |
| Lactobacillus ruminis and Pediococcus acidilactici | -0.490 | 0.046 |
| Fasting insulin |  |  |
| Lactobacillus ruminis and Pediococcus acidilactici | -0.576 | 0.016 |
| Shigella spp. and Echerichia spp. | -0.736 | <0.001 |
| HOMA-IR |  |  |
| Faecalibacterium prausnitzii | -0.559 | 0.020 |
| Lactobacillus ruminis and Pediococcus acidilactici | -0.622 | 0.008 |
| Shigella spp. and Echerichia spp. | -0.551 | 0.022 |
| Matsuda Index |  |  |
| Dorea spp. | -0.493 | 0.045 |
| Lactobacillus ruminis and Pediococcus acidilactici | 0.603 | 0.010 |
| Shigella spp. and Echerichia spp. | 0.545 | 0.024 |
| Lactate |  |  |
| Shigella spp. and Echerichia spp. | -0.496 | 0.043 |
| Citrate |  |  |
| Clostridia. | -0.525 | 0.031 |
| Clostridium sp. | -0.532 | 0.028 |
| Eubacterium rectale | 0.487 | 0.047 |
| Streptococcus agalactiae and Eubacterium rectale | 0.511 | 0.036 |
| Firmicutes (various) | 0.573 | 0.016 |
| Triglycerides |  |  |
| Bacteroides pectinophilus | 0.581 | 0.014 |
| Dialister invisus and Megasphaera micronuciformis | -0.505 | 0.039 |
| Veillonella spp. | -0.510 | 0.037 |
| Shigella spp. and Echerichia spp. | -0.771 | <0.001 |
| NEFA |  |  |
| Bacteroides spp. | 0.732 | <0.001 |
| Clostridium sp. | -0.566 | 0.018 |
| Streptococcus spp. | 0.554 | 0.021 |
| Streptococcus spp. 2 | 0.647 | 0.005 |
| SCFA |  |  |
| Ruminococcus albus and Ruminococcus bromii | 0.667 | 0.003 |
| Mycoplasma hominis | 0.806 | <0.001 |
| Butyrate |  |  |
| Dorea spp. | 0.496 | 0.043 |
| Acetate |  |  |
| Ruminococcus albus and Ruminococcus bromii | 0.625 | 0.007 |
| Streptococcus agalactiae and Eubacterium rectale | -0.490 | 0.046 |
| Mycoplasma hominis | 0.785 | <0.001 |
| Propionate |  |  |
| Actinomycetales | 0.490 | 0.046 |
| Clostridium methylpentosum | -0.500 | 0.041 |

BMI: Body mass index, MVPA: Moderate to vigorous physical activity, NEFA: Non-esterified fatty acids, SCFA: Short chain fatty acid, HbA1c: Glycated hemoglobin
